# 7,7′-(3,3′-Dibenzyl-3*H*,3′*H*-4,4′-bi-1,2,3-triazole-5,5′-di­yl)bis­(4-methyl-2*H*-chromen-2-one)

**DOI:** 10.1107/S1600536808028250

**Published:** 2008-09-13

**Authors:** Jessie A. Key, Christopher W. Cairo, Michael J. Ferguson

**Affiliations:** a Alberta Ingenuity Centre for Carbohydrate Science, Department of Chemistry, University of Alberta, Edmonton, Alberta, Canada T6G 2G2; b X-ray Crystallography Laboratory, Department of Chemistry, University of Alberta, Edmonton, Alberta, Canada T6G 2G2

## Abstract

The title compound, a bis-5,5′-triazole, C_38_H_28_N_6_O_4_, was observed as a side-product from the Sharpless–Meldal click reaction of the corresponding coumarin alkyne and benzyl­azide. Although the compound was present as a minor component, it crystallized in preference to the major product. The two triazole rings are almost orthogonal to each other [dihedral angle = 83.8 (1)°]. However the 4 and 4′ coumarin systems are close to coplanar with their respective triazole rings [23.6 (1) and 15.1 (1)°]. Each of the benzene rings packs approximately face-to-face with the opposing coumarin ring systems, with inter­planar angles of 7.7 (1) and 25.3 (1)° and distances of 3.567 (2) and 3.929 (2) Å between the respective centroids of the opposing rings.

## Related literature

Similar 5,5′-bis­triazole structures have been described previously by Angell & Burgess (2007 ▶). For the synthesis of related alkyne-modified coumarins, see: Sivakumar *et al.* (2004 ▶); Zhou & Fahrni (2004 ▶).
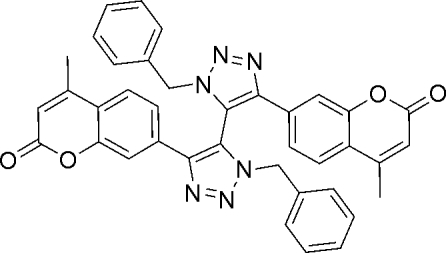


## Experimental

### 

#### Crystal data

C_38_H_28_N_6_O_4_
*M* *_r_* = 632.66
Monoclinic, 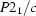

*a* = 12.4328 (17) Å
*b* = 17.565 (2) Å
*c* = 14.456 (2) Å
β = 94.573 (3)°
*V* = 3147.0 (7) Å^3^
*Z* = 4
Mo *K*α radiation
μ = 0.09 mm^−1^
*T* = 193 (2) K
0.36 × 0.19 × 0.06 mm

#### Data collection

Bruker SMART 1000 CCD diffractometer
Absorption correction: multi-scan (*SADABS*; Sheldrick, 2003 ▶) *T*_min_ = 0.969, *T*_max_ = 0.995
21410 measured reflections
5703 independent reflections
3222 reflections with *I* > 2σ(*I*)
*R*_int_ = 0.074

#### Refinement

*R*[*F* ^2^ > 2σ(*F* ^2^)] = 0.048
*wR*(*F* ^2^) = 0.114
*S* = 1.00
5703 reflections
435 parameters
H-atom parameters constrained
Δρ_max_ = 0.15 e Å^−3^
Δρ_min_ = −0.16 e Å^−3^

### 

Data collection: *SMART* (Bruker, 1997 ▶); cell refinement: *SAINT* (Bruker, 1997 ▶); data reduction: *SAINT*; program(s) used to solve structure: *SHELXS97* (Sheldrick, 2008 ▶); program(s) used to refine structure: *SHELXL97* (Sheldrick, 2008 ▶); molecular graphics: *SHELXTL* (Sheldrick, 2008 ▶); software used to prepare material for publication: *SHELXTL*.

## Supplementary Material

Crystal structure: contains datablocks I, global. DOI: 10.1107/S1600536808028250/fb2110sup1.cif

Structure factors: contains datablocks I. DOI: 10.1107/S1600536808028250/fb2110Isup2.hkl

Additional supplementary materials:  crystallographic information; 3D view; checkCIF report

## Figures and Tables

**Table 1:** Hydrogen-bond geometry (Å, °)

*D*—H⋯*A*	*D*—H	H⋯*A*	*D*⋯*A*	*D*—H⋯*A*
C5—H5⋯O2^i^	0.95	2.45	3.292 (3)	148
C33—H33*B*⋯O2^ii^	0.99	2.33	3.307 (3)	168
C10—H10*C*⋯O4^iii^	0.98	2.52	3.337 (4)	141

Symmetry codes: (i) 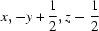; (ii) 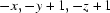; (iii) 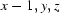.

**Fig. 1.**
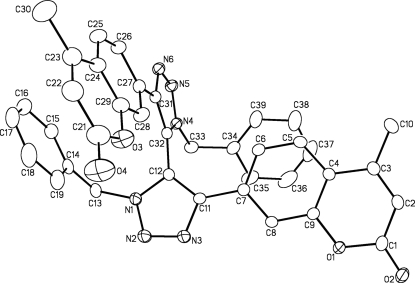
Perspective view of (I) showing the atom labelling scheme. Non-hydrogen atoms are represented by Gaussian ellipsoids at the 20% probability level. Hydrogen atoms are not shown.

**Fig. 2.**
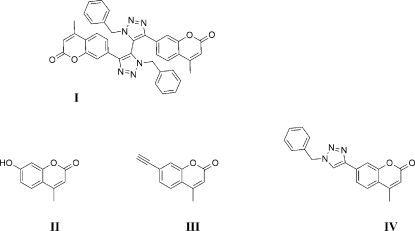
Compounds used in this study.

## supplementary crystallographic information

### Comment

In our studies of new synthetic fluorophores through modification of a common fluorophore structure 4-methyl-unbelliferone (II), we generated new alkyne containing profluorophores. We subjected alkyne-modified coumarin structure (III) to conditions typical in a Sharpless–Meldal click reaction with the intention of forming the corresponding 1,2,3-triazole (IV). We explored several conditions for the synthesis of (IV), and obtained reasonable yields with (III) and benzyl azide when reacted with CuI and TEA in a methanol:water mixture. In some of these reactions we observed a minor side-product (23%) evidenced by the appearance of two doublet peaks between 4–5 ppm in the ^1^H NMR spectrum. These resonances were attributed to the benzylic hydrogen atoms of a bis-5,5'-triazole structure (I), and the presence of this side product was confirmed by the accompanying crystal structure data. This type of side product was first reported by Angell & Burgess (2007). Those authors reported similar observations by ^1^H NMR and crystallography of the bis-triazole adduct. We have identified improved conditions that avoid formation of the bis-triazole, however it is notable that commonly used conditions for click reactions may produce this type of side product.

### Experimental

Synthesis of triazole (IV): The alkyne, (III) (1 equiv), and benzyl azide (4–5 equiv) were dissolved in a 1:1 solution of methanol:water (0.03 *M* alkyne). CuI (0.2 equiv) was then added, followed by triethylamine (TEA) (2 equiv). The reaction proceeded at room temperature and was monitored by thin layer chromatography. The reaction was complete within 2–3 h. The crude product was concentrated *in vacuo*, extracted with chloroform and purified by flash column chromatography (CH_2_Cl_2_/MeOH), a small amount of the bis-5,5'-triazole (I) was present (23%). The mixture of I and IV was dissolved in 200 µl chloroform, followed by 800 µl of hexanes. Suitable crystals were obtained after two weeks. The crystals were used for determination of the X-ray structure. The original product mixture, 77:23 of IV and I, was used for NMR and MS analysis. ^1^H NMR (400 MHz, CDCl_3_):** δ 7.85 (dd, 1H, ^3^J = 10.8 Hz, ^4^J = 2.1 Hz), 7.76 (s, 1H), 7.67 (d, 1H, ^4^J = 2.1 Hz), 7.64 (d, 1H, ^3^J = 10.8 Hz), 7.43- 7.32 (m, 7H), 7.22 (d, 1H, ^4^J = 1.6 Hz), 7.05 (m, 2H)^I^, 6.68 (m, 1H)^I^, 6.29 (d, 1H, ^4^J = 1 Hz), 6.26 (d, 0.5H, ^4^J = 1.6 Hz), 5.61 (s, 2H), 4.88 (d, 0.7H, ^3^J = 15.2 Hz)^I^, 4.63 (d, 0.7H, ^3^J = 15.2 Hz)^I^, 2.45 (s, 3H), 2.37 (s, 1.5H)^I^. APT ^13^C NMR (100 MHz, CDCl_3_): δ 160.7, 160.3, 153.9, 153.5, 152.0, 151.6, 134.3, 132.6, 132.3, 129.3, 129.0, 128.8, 128.6, 128.2, 128.0, 125.2, 121.5, 121.3, 120.1, 119.6, 115.5, 115.0, 113.8, 113.6, 54.5, 53.0, 18.6, 18.5. HRMS calculated for C_38_H_28_N_6_O_4_: 632.22; observed: 632.21768 ([2*M*-2H]^+^). HRMS calculated for C_19_H_15_N_3_O_2_: 317.12; observed: 340.11635 ([*M*+Na]^+^). *Rf* = 0.68 (10:1 CH_2_Cl_2_/MeOH). **NMR peaks attributed to compound I are denoted by a superscript, and were not observed in purified samples of IV.

### Refinement

All the hydrogen atoms could have been discerned in the difference electron density map, nevertheless, all the H atoms were generated in idealized positions and then refined using a riding model with fixed C—H distances (C_aryl_ = 0.95 Å, C_methyl_ = 0.98 Å, C_methylene_ = 0.99 Å) and with *U*_iso_(H) = 1.2*U*_eq_(C).

### Crystal data

**Table:**

C_38_H_28_N_6_O_4_	*F*(000) = 1320
*M**_r_* = 632.66	*D*_x_ = 1.335 Mg m^−^^3^
Monoclinic, *P*2_1_/*c*	Mo *K*α radiation, λ = 0.71073 Å
Hall symbol: -P 2ybc	Cell parameters from 2863 reflections
*a* = 12.4328 (17) Å	θ = 2.3–20.4°
*b* = 17.565 (2) Å	µ = 0.09 mm^−^^1^
*c* = 14.456 (2) Å	*T* = 193 K
β = 94.573 (3)°	Plate, colourless
*V* = 3147.0 (7) Å^3^	0.36 × 0.19 × 0.06 mm
*Z* = 4	

### Data collection

**Table:**

Bruker PLATFORM diffractometer/SMART 1000 CCD area-detector	5703 independent reflections
Radiation source: fine-focus sealed tube	3222 reflections with *I* > 2σ(*I*)
graphite	*R*_int_ = 0.074
Detector resolution: 8.192 pixels mm^-1^	θ_max_ = 25.3°, θ_min_ = 1.6°
ω scans	*h* = −14→14
Absorption correction: multi-scan (SADABS; Sheldrick, 2003)	*k* = −21→21
*T*_min_ = 0.969, *T*_max_ = 0.995	*l* = −17→17
21410 measured reflections	

### Refinement

**Table:**

Refinement on *F*^2^	Primary atom site location: structure-invariant direct methods
Least-squares matrix: full	Secondary atom site location: difference Fourier map
*R*[*F*^2^ > 2σ(*F*^2^)] = 0.048	Hydrogen site location: difference Fourier map
*wR*(*F*^2^) = 0.114	H-atom parameters constrained
*S* = 1.00	*w* = 1/[σ^2^(*F*_o_^2^) + (0.0383*P*)^2^ + 0.7089*P*] where *P* = (*F*_o_^2^ + 2*F*_c_^2^)/3
5703 reflections	(Δ/σ)_max_ < 0.001
435 parameters	Δρ_max_ = 0.15 e Å^−^^3^
0 restraints	Δρ_min_ = −0.16 e Å^−^^3^
110 constraints	

### Special details

**Table:**

Geometry. All e.s.d.'s (except the e.s.d. in the dihedral angle between two l.s. planes) are estimated using the full covariance matrix. The cell e.s.d.'s are taken into account individually in the estimation of e.s.d.'s in distances, angles and torsion angles; correlations between e.s.d.'s in cell parameters are only used when they are defined by crystal symmetry. An approximate (isotropic) treatment of cell e.s.d.'s is used for estimating e.s.d.'s involving l.s. planes.
Refinement. Refinement of *F*^2^ against ALL reflections. The weighted *R*-factor *wR* and goodness of fit *S* are based on *F*^2^, conventional *R*-factors *R* are based on *F*, with *F* set to zero for negative *F*^2^. The threshold expression of *F*^2^ > σ(*F*^2^) is used only for calculating *R*-factors(gt) *etc*. and is not relevant to the choice of reflections for refinement. *R*-factors based on *F*^2^ are statistically about twice as large as those based on *F*, and *R*- factors based on ALL data will be even larger.

### Fractional atomic coordinates and isotropic or equivalent isotropic displacement parameters (Å^2^)

**Table:**

	*x*	*y*	*z*	*U*_iso_*/*U*_eq_	
O1	−0.04554 (13)	0.38038 (9)	0.61080 (10)	0.0487 (4)	
O2	−0.11506 (15)	0.35827 (9)	0.74362 (12)	0.0611 (5)	
O3	0.40923 (16)	0.24278 (11)	0.23341 (13)	0.0765 (6)	
O4	0.5627 (2)	0.20011 (15)	0.29991 (18)	0.1143 (9)	
N1	0.22902 (15)	0.49591 (11)	0.24171 (13)	0.0435 (5)	
N2	0.27468 (17)	0.50775 (12)	0.32747 (14)	0.0529 (6)	
N3	0.21771 (16)	0.47016 (11)	0.38566 (13)	0.0493 (5)	
N4	−0.01392 (15)	0.46189 (10)	0.12514 (12)	0.0399 (5)	
N5	−0.05450 (16)	0.42958 (11)	0.04549 (13)	0.0460 (5)	
N6	0.00981 (16)	0.37304 (11)	0.02675 (13)	0.0441 (5)	
C1	−0.1166 (2)	0.34162 (14)	0.66209 (18)	0.0479 (6)	
C2	−0.18458 (19)	0.28631 (13)	0.61442 (17)	0.0471 (6)	
H2	−0.2375	0.2616	0.6476	0.056*	
C3	−0.17758 (19)	0.26742 (13)	0.52525 (17)	0.0431 (6)	
C4	−0.09753 (18)	0.30559 (12)	0.47449 (15)	0.0389 (6)	
C5	−0.0778 (2)	0.28938 (13)	0.38278 (16)	0.0466 (6)	
H5	−0.1172	0.2498	0.3507	0.056*	
C6	−0.00257 (19)	0.32950 (13)	0.33810 (16)	0.0455 (6)	
H6	0.0097	0.3171	0.2759	0.055*	
C7	0.05606 (18)	0.38843 (12)	0.38319 (15)	0.0381 (6)	
C8	0.03910 (18)	0.40452 (12)	0.47489 (15)	0.0407 (6)	
H8	0.0785	0.4440	0.5071	0.049*	
C9	−0.03566 (18)	0.36255 (13)	0.51869 (15)	0.0392 (6)	
C10	−0.2493 (2)	0.20886 (15)	0.47772 (18)	0.0610 (8)	
H10A	−0.2972	0.1877	0.5219	0.073*	
H10B	−0.2052	0.1680	0.4543	0.073*	
H10C	−0.2927	0.2324	0.4258	0.073*	
C11	0.13516 (18)	0.43373 (12)	0.33611 (15)	0.0392 (6)	
C12	0.14149 (18)	0.44964 (12)	0.24347 (15)	0.0368 (5)	
C13	0.27160 (19)	0.53422 (14)	0.16239 (16)	0.0487 (7)	
H13A	0.3156	0.5784	0.1852	0.058*	
H13B	0.2105	0.5539	0.1211	0.058*	
C14	0.33957 (19)	0.48340 (14)	0.10704 (18)	0.0476 (6)	
C15	0.3150 (2)	0.47500 (15)	0.01346 (18)	0.0556 (7)	
H15	0.2546	0.5013	−0.0155	0.067*	
C16	0.3761 (2)	0.42923 (18)	−0.0396 (2)	0.0715 (9)	
H16	0.3577	0.4244	−0.1044	0.086*	
C17	0.4629 (3)	0.39096 (18)	0.0011 (3)	0.0779 (10)	
H17	0.5043	0.3586	−0.0349	0.094*	
C18	0.4899 (2)	0.3996 (2)	0.0946 (3)	0.0861 (10)	
H18	0.5512	0.3738	0.1228	0.103*	
C19	0.4289 (2)	0.44567 (18)	0.1482 (2)	0.0715 (9)	
H19	0.4482	0.4513	0.2128	0.086*	
C21	0.5019 (3)	0.19979 (19)	0.2306 (3)	0.0810 (10)	
C22	0.5171 (2)	0.16063 (18)	0.1459 (3)	0.0800 (10)	
H22	0.5796	0.1299	0.1431	0.096*	
C23	0.4479 (2)	0.16503 (17)	0.0704 (2)	0.0717 (9)	
C24	0.3521 (2)	0.21249 (15)	0.07429 (19)	0.0562 (7)	
C25	0.2752 (2)	0.22437 (16)	0.0006 (2)	0.0631 (8)	
H25	0.2817	0.1980	−0.0561	0.076*	
C26	0.1900 (2)	0.27323 (14)	0.00755 (18)	0.0528 (7)	
H26	0.1389	0.2806	−0.0441	0.063*	
C27	0.17829 (19)	0.31216 (13)	0.09072 (16)	0.0431 (6)	
C28	0.2519 (2)	0.29845 (14)	0.16581 (17)	0.0512 (7)	
H28	0.2434	0.3224	0.2237	0.061*	
C29	0.3372 (2)	0.25008 (15)	0.15623 (19)	0.0537 (7)	
C30	0.4669 (3)	0.1241 (2)	−0.0173 (2)	0.1060 (13)	
H30A	0.5311	0.0919	−0.0072	0.127*	
H30B	0.4779	0.1613	−0.0663	0.127*	
H30C	0.4042	0.0924	−0.0361	0.127*	
C31	0.09201 (18)	0.36899 (13)	0.09483 (15)	0.0382 (6)	
C32	0.07816 (17)	0.42608 (12)	0.15855 (15)	0.0364 (5)	
C33	−0.07212 (19)	0.52408 (13)	0.16521 (17)	0.0481 (6)	
H33A	−0.1075	0.5553	0.1145	0.058*	
H33B	−0.0200	0.5570	0.2018	0.058*	
C34	−0.1562 (2)	0.49688 (16)	0.22682 (16)	0.0492 (7)	
C35	−0.1863 (3)	0.5442 (2)	0.2960 (2)	0.0877 (11)	
H35	−0.1547	0.5933	0.3036	0.105*	
C36	−0.2617 (3)	0.5209 (3)	0.3541 (3)	0.1298 (18)	
H36	−0.2814	0.5536	0.4022	0.156*	
C37	−0.3088 (3)	0.4506 (3)	0.3432 (3)	0.1210 (17)	
H37	−0.3612	0.4349	0.3837	0.145*	
C38	−0.2809 (3)	0.4032 (2)	0.2748 (2)	0.0917 (11)	
H38	−0.3137	0.3546	0.2667	0.110*	
C39	−0.2042 (2)	0.42693 (18)	0.2171 (2)	0.0676 (8)	
H39	−0.1842	0.3938	0.1694	0.081*	

### Atomic displacement parameters (Å^2^)

**Table:**

	*U*^11^	*U*^22^	*U*^33^	*U*^12^	*U*^13^	*U*^23^
O1	0.0625 (12)	0.0467 (10)	0.0383 (10)	−0.0123 (9)	0.0128 (9)	−0.0055 (8)
O2	0.0865 (14)	0.0564 (11)	0.0424 (11)	−0.0080 (10)	0.0177 (10)	−0.0004 (9)
O3	0.0629 (13)	0.0901 (15)	0.0732 (14)	0.0306 (11)	−0.0150 (11)	−0.0122 (11)
O4	0.0845 (18)	0.139 (2)	0.112 (2)	0.0492 (16)	−0.0406 (16)	−0.0220 (16)
N1	0.0425 (12)	0.0502 (12)	0.0374 (12)	−0.0082 (10)	0.0017 (10)	0.0042 (10)
N2	0.0550 (14)	0.0602 (14)	0.0424 (13)	−0.0147 (11)	−0.0027 (11)	0.0027 (11)
N3	0.0523 (13)	0.0551 (13)	0.0399 (12)	−0.0127 (11)	0.0001 (11)	0.0015 (10)
N4	0.0404 (12)	0.0449 (12)	0.0346 (11)	0.0009 (10)	0.0040 (9)	−0.0021 (9)
N5	0.0431 (12)	0.0550 (13)	0.0394 (12)	0.0023 (11)	0.0005 (10)	−0.0043 (10)
N6	0.0412 (12)	0.0510 (13)	0.0402 (12)	0.0007 (10)	0.0041 (10)	−0.0037 (10)
C1	0.0574 (17)	0.0426 (15)	0.0453 (16)	0.0012 (13)	0.0138 (14)	0.0065 (13)
C2	0.0447 (15)	0.0447 (15)	0.0531 (17)	−0.0056 (12)	0.0114 (13)	0.0038 (13)
C3	0.0409 (15)	0.0391 (14)	0.0489 (16)	−0.0003 (11)	0.0011 (13)	0.0037 (12)
C4	0.0406 (14)	0.0353 (13)	0.0404 (15)	−0.0026 (11)	0.0015 (12)	0.0006 (11)
C5	0.0547 (17)	0.0436 (14)	0.0410 (15)	−0.0109 (13)	−0.0008 (13)	−0.0043 (12)
C6	0.0561 (16)	0.0476 (15)	0.0330 (14)	−0.0081 (13)	0.0046 (12)	−0.0033 (12)
C7	0.0397 (14)	0.0379 (14)	0.0363 (14)	0.0004 (11)	0.0012 (11)	0.0023 (11)
C8	0.0479 (15)	0.0370 (13)	0.0372 (14)	−0.0084 (11)	0.0026 (12)	−0.0043 (11)
C9	0.0446 (15)	0.0386 (14)	0.0345 (14)	0.0011 (12)	0.0041 (12)	−0.0010 (11)
C10	0.0523 (17)	0.0643 (18)	0.0657 (19)	−0.0200 (14)	0.0005 (14)	0.0008 (15)
C11	0.0421 (14)	0.0406 (14)	0.0344 (14)	−0.0030 (11)	0.0007 (12)	−0.0031 (11)
C12	0.0360 (14)	0.0363 (13)	0.0381 (14)	−0.0015 (11)	0.0036 (11)	0.0005 (11)
C13	0.0465 (15)	0.0547 (16)	0.0452 (15)	−0.0101 (13)	0.0066 (12)	0.0112 (12)
C14	0.0345 (14)	0.0573 (17)	0.0516 (17)	−0.0077 (12)	0.0069 (13)	0.0111 (13)
C15	0.0486 (17)	0.0635 (18)	0.0550 (18)	−0.0015 (14)	0.0052 (14)	0.0027 (14)
C16	0.065 (2)	0.085 (2)	0.066 (2)	−0.0007 (18)	0.0158 (17)	−0.0099 (17)
C17	0.063 (2)	0.078 (2)	0.098 (3)	0.0030 (18)	0.038 (2)	0.003 (2)
C18	0.050 (2)	0.105 (3)	0.105 (3)	0.0202 (18)	0.020 (2)	0.028 (2)
C19	0.0469 (18)	0.102 (2)	0.066 (2)	0.0033 (17)	0.0075 (16)	0.0221 (18)
C21	0.056 (2)	0.090 (2)	0.094 (3)	0.0247 (18)	−0.015 (2)	−0.013 (2)
C22	0.0467 (19)	0.085 (2)	0.107 (3)	0.0192 (17)	0.002 (2)	−0.019 (2)
C23	0.0444 (18)	0.078 (2)	0.093 (2)	0.0118 (16)	0.0065 (18)	−0.0228 (18)
C24	0.0391 (16)	0.0618 (18)	0.0676 (19)	0.0048 (13)	0.0034 (15)	−0.0201 (15)
C25	0.0468 (17)	0.078 (2)	0.0645 (19)	0.0038 (15)	0.0040 (15)	−0.0303 (16)
C26	0.0409 (16)	0.0644 (18)	0.0525 (16)	0.0019 (13)	0.0008 (13)	−0.0155 (14)
C27	0.0366 (14)	0.0470 (15)	0.0465 (16)	−0.0010 (12)	0.0077 (12)	−0.0043 (12)
C28	0.0515 (17)	0.0579 (17)	0.0440 (16)	0.0123 (14)	0.0022 (13)	−0.0056 (13)
C29	0.0421 (16)	0.0593 (17)	0.0580 (17)	0.0070 (14)	−0.0062 (14)	−0.0075 (14)
C30	0.067 (2)	0.133 (3)	0.119 (3)	0.039 (2)	0.010 (2)	−0.050 (3)
C31	0.0336 (13)	0.0461 (14)	0.0349 (13)	−0.0026 (11)	0.0030 (12)	0.0006 (11)
C32	0.0331 (13)	0.0413 (14)	0.0348 (13)	−0.0030 (11)	0.0035 (11)	0.0029 (11)
C33	0.0495 (16)	0.0463 (15)	0.0480 (15)	0.0070 (12)	−0.0002 (13)	−0.0058 (12)
C34	0.0443 (15)	0.0636 (18)	0.0394 (15)	0.0143 (14)	0.0004 (12)	0.0009 (13)
C35	0.064 (2)	0.127 (3)	0.074 (2)	0.009 (2)	0.0146 (18)	−0.041 (2)
C36	0.081 (3)	0.230 (6)	0.082 (3)	0.004 (3)	0.032 (2)	−0.061 (3)
C37	0.073 (3)	0.220 (6)	0.075 (3)	−0.006 (3)	0.037 (2)	0.009 (3)
C38	0.071 (2)	0.123 (3)	0.085 (3)	0.004 (2)	0.029 (2)	0.025 (2)
C39	0.065 (2)	0.074 (2)	0.067 (2)	0.0103 (17)	0.0268 (16)	0.0103 (16)

### Geometric parameters (Å, °)

**Table:**

O1—C1	1.378 (3)	C15—H15	0.9500
O1—C9	1.383 (2)	C16—C17	1.364 (4)
O2—C1	1.213 (3)	C16—H16	0.9500
O3—C29	1.380 (3)	C17—C18	1.374 (4)
O3—C21	1.381 (3)	C17—H17	0.9500
O4—C21	1.206 (3)	C18—C19	1.388 (4)
N1—N2	1.338 (2)	C18—H18	0.9500
N1—C12	1.360 (3)	C19—H19	0.9500
N1—C13	1.465 (3)	C21—C22	1.431 (4)
N2—N3	1.319 (2)	C22—C23	1.337 (4)
N3—C11	1.363 (3)	C22—H22	0.9500
N4—N5	1.345 (2)	C23—C24	1.459 (4)
N4—C32	1.361 (3)	C23—C30	1.493 (4)
N4—C33	1.456 (3)	C24—C29	1.381 (3)
N5—N6	1.317 (2)	C24—C25	1.390 (3)
N6—C31	1.363 (3)	C25—C26	1.374 (3)
C1—C2	1.428 (3)	C25—H25	0.9500
C2—C3	1.341 (3)	C26—C27	1.401 (3)
C2—H2	0.9500	C26—H26	0.9500
C3—C4	1.447 (3)	C27—C28	1.384 (3)
C3—C10	1.493 (3)	C27—C31	1.470 (3)
C4—C9	1.387 (3)	C28—C29	1.375 (3)
C4—C5	1.397 (3)	C28—H28	0.9500
C5—C6	1.373 (3)	C30—H30A	0.9800
C5—H5	0.9500	C30—H30B	0.9800
C6—C7	1.397 (3)	C30—H30C	0.9800
C6—H6	0.9500	C31—C32	1.382 (3)
C7—C8	1.388 (3)	C33—C34	1.504 (3)
C7—C11	1.473 (3)	C33—H33A	0.9900
C8—C9	1.379 (3)	C33—H33B	0.9900
C8—H8	0.9500	C34—C39	1.368 (4)
C10—H10A	0.9800	C34—C35	1.376 (4)
C10—H10B	0.9800	C35—C36	1.370 (5)
C10—H10C	0.9800	C35—H35	0.9500
C11—C12	1.376 (3)	C36—C37	1.371 (6)
C12—C32	1.464 (3)	C36—H36	0.9500
C13—C14	1.503 (3)	C37—C38	1.359 (5)
C13—H13A	0.9900	C37—H37	0.9500
C13—H13B	0.9900	C38—C39	1.381 (4)
C14—C15	1.371 (3)	C38—H38	0.9500
C14—C19	1.387 (4)	C39—H39	0.9500
C15—C16	1.380 (4)		
			
C1—O1—C9	121.08 (19)	C17—C18—C19	120.8 (3)
C29—O3—C21	121.1 (2)	C17—C18—H18	119.6
N2—N1—C12	110.89 (18)	C19—C18—H18	119.6
N2—N1—C13	120.06 (19)	C14—C19—C18	119.6 (3)
C12—N1—C13	129.0 (2)	C14—C19—H19	120.2
N3—N2—N1	107.66 (18)	C18—C19—H19	120.2
N2—N3—C11	108.65 (18)	O4—C21—O3	116.3 (3)
N5—N4—C32	110.87 (18)	O4—C21—C22	126.7 (3)
N5—N4—C33	119.52 (19)	O3—C21—C22	117.1 (3)
C32—N4—C33	129.53 (19)	C23—C22—C21	123.4 (3)
N6—N5—N4	107.57 (18)	C23—C22—H22	118.3
N5—N6—C31	108.87 (18)	C21—C22—H22	118.3
O2—C1—O1	116.3 (2)	C22—C23—C24	118.4 (3)
O2—C1—C2	126.6 (2)	C22—C23—C30	122.1 (3)
O1—C1—C2	117.2 (2)	C24—C23—C30	119.5 (3)
C3—C2—C1	123.4 (2)	C29—C24—C25	117.1 (2)
C3—C2—H2	118.3	C29—C24—C23	118.2 (3)
C1—C2—H2	118.3	C25—C24—C23	124.8 (3)
C2—C3—C4	118.2 (2)	C26—C25—C24	121.7 (2)
C2—C3—C10	122.1 (2)	C26—C25—H25	119.2
C4—C3—C10	119.7 (2)	C24—C25—H25	119.2
C9—C4—C5	116.8 (2)	C25—C26—C27	120.1 (2)
C9—C4—C3	118.6 (2)	C25—C26—H26	119.9
C5—C4—C3	124.5 (2)	C27—C26—H26	120.0
C6—C5—C4	121.3 (2)	C28—C27—C26	118.8 (2)
C6—C5—H5	119.3	C28—C27—C31	121.7 (2)
C4—C5—H5	119.3	C26—C27—C31	119.5 (2)
C5—C6—C7	120.7 (2)	C29—C28—C27	119.7 (2)
C5—C6—H6	119.7	C29—C28—H28	120.1
C7—C6—H6	119.7	C27—C28—H28	120.1
C8—C7—C6	118.9 (2)	C28—C29—O3	115.6 (2)
C8—C7—C11	119.3 (2)	C28—C29—C24	122.6 (2)
C6—C7—C11	121.8 (2)	O3—C29—C24	121.8 (2)
C9—C8—C7	119.3 (2)	C23—C30—H30A	109.5
C9—C8—H8	120.4	C23—C30—H30B	109.5
C7—C8—H8	120.4	H30A—C30—H30B	109.5
C8—C9—O1	115.8 (2)	C23—C30—H30C	109.5
C8—C9—C4	122.9 (2)	H30A—C30—H30C	109.5
O1—C9—C4	121.2 (2)	H30B—C30—H30C	109.5
C3—C10—H10A	109.5	N6—C31—C32	108.5 (2)
C3—C10—H10B	109.5	N6—C31—C27	120.9 (2)
H10A—C10—H10B	109.5	C32—C31—C27	130.5 (2)
C3—C10—H10C	109.5	N4—C32—C31	104.18 (19)
H10A—C10—H10C	109.5	N4—C32—C12	123.2 (2)
H10B—C10—H10C	109.5	C31—C32—C12	132.6 (2)
N3—C11—C12	108.5 (2)	N4—C33—C34	112.85 (19)
N3—C11—C7	120.9 (2)	N4—C33—H33A	109.0
C12—C11—C7	130.6 (2)	C34—C33—H33A	109.0
N1—C12—C11	104.33 (19)	N4—C33—H33B	109.0
N1—C12—C32	122.06 (19)	C34—C33—H33B	109.0
C11—C12—C32	133.6 (2)	H33A—C33—H33B	107.8
N1—C13—C14	113.43 (19)	C39—C34—C35	118.4 (3)
N1—C13—H13A	108.9	C39—C34—C33	122.8 (2)
C14—C13—H13A	108.9	C35—C34—C33	118.7 (3)
N1—C13—H13B	108.9	C36—C35—C34	120.2 (4)
C14—C13—H13B	108.9	C36—C35—H35	119.9
H13A—C13—H13B	107.7	C34—C35—H35	119.9
C15—C14—C19	118.7 (3)	C35—C36—C37	120.5 (4)
C15—C14—C13	119.9 (2)	C35—C36—H36	119.8
C19—C14—C13	121.4 (2)	C37—C36—H36	119.8
C14—C15—C16	121.5 (3)	C38—C37—C36	120.2 (4)
C14—C15—H15	119.3	C38—C37—H37	119.9
C16—C15—H15	119.3	C36—C37—H37	119.9
C17—C16—C15	119.9 (3)	C37—C38—C39	119.0 (4)
C17—C16—H16	120.0	C37—C38—H38	120.5
C15—C16—H16	120.0	C39—C38—H38	120.5
C16—C17—C18	119.5 (3)	C34—C39—C38	121.7 (3)
C16—C17—H17	120.3	C34—C39—H39	119.2
C18—C17—H17	120.3	C38—C39—H39	119.2
			
C12—N1—N2—N3	−0.4 (3)	C17—C18—C19—C14	0.0 (5)
C13—N1—N2—N3	176.64 (19)	C29—O3—C21—O4	176.2 (3)
N1—N2—N3—C11	0.3 (3)	C29—O3—C21—C22	−2.8 (4)
C32—N4—N5—N6	0.2 (2)	O4—C21—C22—C23	−177.5 (4)
C33—N4—N5—N6	−176.94 (18)	O3—C21—C22—C23	1.4 (5)
N4—N5—N6—C31	0.1 (2)	C21—C22—C23—C24	0.3 (5)
C9—O1—C1—O2	175.8 (2)	C21—C22—C23—C30	179.2 (3)
C9—O1—C1—C2	−4.3 (3)	C22—C23—C24—C29	−0.5 (4)
O2—C1—C2—C3	−175.8 (3)	C30—C23—C24—C29	−179.5 (3)
O1—C1—C2—C3	4.3 (4)	C22—C23—C24—C25	177.9 (3)
C1—C2—C3—C4	−0.5 (4)	C30—C23—C24—C25	−1.1 (5)
C1—C2—C3—C10	179.5 (2)	C29—C24—C25—C26	2.1 (4)
C2—C3—C4—C9	−3.3 (3)	C23—C24—C25—C26	−176.3 (3)
C10—C3—C4—C9	176.7 (2)	C24—C25—C26—C27	−0.5 (4)
C2—C3—C4—C5	177.0 (2)	C25—C26—C27—C28	−2.1 (4)
C10—C3—C4—C5	−3.0 (4)	C25—C26—C27—C31	174.9 (2)
C9—C4—C5—C6	−1.6 (3)	C26—C27—C28—C29	3.1 (4)
C3—C4—C5—C6	178.1 (2)	C31—C27—C28—C29	−173.9 (2)
C4—C5—C6—C7	−0.5 (4)	C27—C28—C29—O3	177.0 (2)
C5—C6—C7—C8	1.7 (3)	C27—C28—C29—C24	−1.5 (4)
C5—C6—C7—C11	−178.3 (2)	C21—O3—C29—C28	−175.8 (3)
C6—C7—C8—C9	−0.6 (3)	C21—O3—C29—C24	2.7 (4)
C11—C7—C8—C9	179.4 (2)	C25—C24—C29—C28	−1.1 (4)
C7—C8—C9—O1	178.1 (2)	C23—C24—C29—C28	177.4 (3)
C7—C8—C9—C4	−1.7 (3)	C25—C24—C29—O3	−179.4 (2)
C1—O1—C9—C8	−179.2 (2)	C23—C24—C29—O3	−1.0 (4)
C1—O1—C9—C4	0.6 (3)	N5—N6—C31—C32	−0.3 (2)
C5—C4—C9—C8	2.8 (3)	N5—N6—C31—C27	−178.29 (19)
C3—C4—C9—C8	−177.0 (2)	C28—C27—C31—N6	−168.9 (2)
C5—C4—C9—O1	−176.9 (2)	C26—C27—C31—N6	14.2 (3)
C3—C4—C9—O1	3.3 (3)	C28—C27—C31—C32	13.6 (4)
N2—N3—C11—C12	−0.1 (3)	C26—C27—C31—C32	−163.3 (2)
N2—N3—C11—C7	−178.5 (2)	N5—N4—C32—C31	−0.4 (2)
C8—C7—C11—N3	24.2 (3)	C33—N4—C32—C31	176.4 (2)
C6—C7—C11—N3	−155.8 (2)	N5—N4—C32—C12	178.81 (19)
C8—C7—C11—C12	−153.9 (2)	C33—N4—C32—C12	−4.4 (3)
C6—C7—C11—C12	26.2 (4)	N6—C31—C32—N4	0.4 (2)
N2—N1—C12—C11	0.3 (2)	C27—C31—C32—N4	178.1 (2)
C13—N1—C12—C11	−176.4 (2)	N6—C31—C32—C12	−178.6 (2)
N2—N1—C12—C32	−178.5 (2)	C27—C31—C32—C12	−0.9 (4)
C13—N1—C12—C32	4.8 (3)	N1—C12—C32—N4	−96.3 (3)
N3—C11—C12—N1	−0.2 (2)	C11—C12—C32—N4	85.2 (3)
C7—C11—C12—N1	178.1 (2)	N1—C12—C32—C31	82.6 (3)
N3—C11—C12—C32	178.5 (2)	C11—C12—C32—C31	−95.9 (3)
C7—C11—C12—C32	−3.3 (4)	N5—N4—C33—C34	86.5 (2)
N2—N1—C13—C14	102.5 (2)	C32—N4—C33—C34	−90.0 (3)
C12—N1—C13—C14	−81.1 (3)	N4—C33—C34—C39	−25.5 (3)
N1—C13—C14—C15	125.2 (2)	N4—C33—C34—C35	154.5 (2)
N1—C13—C14—C19	−56.2 (3)	C39—C34—C35—C36	0.8 (5)
C19—C14—C15—C16	1.0 (4)	C33—C34—C35—C36	−179.2 (3)
C13—C14—C15—C16	179.5 (2)	C34—C35—C36—C37	−0.8 (6)
C14—C15—C16—C17	0.2 (4)	C35—C36—C37—C38	0.2 (7)
C15—C16—C17—C18	−1.3 (5)	C36—C37—C38—C39	0.4 (6)
C16—C17—C18—C19	1.2 (5)	C35—C34—C39—C38	−0.2 (4)
C15—C14—C19—C18	−1.1 (4)	C33—C34—C39—C38	179.8 (3)
C13—C14—C19—C18	−179.6 (2)	C37—C38—C39—C34	−0.4 (5)

### Hydrogen-bond geometry (Å, °)

**Table:**

*D*—H···*A*	*D*—H	H···*A*	*D*···*A*	*D*—H···*A*
C5—H5···O2^i^	0.95	2.45	3.292 (3)	148.
C33—H33B···O2^ii^	0.99	2.33	3.307 (3)	168.
C10—H10C···O4^iii^	0.98	2.52	3.337 (4)	141.

Symmetry codes: (i) *x*, −*y*+1/2, *z*−1/2; (ii) −*x*, −*y*+1, −*z*+1; (iii) *x*−1, *y*, *z*.
